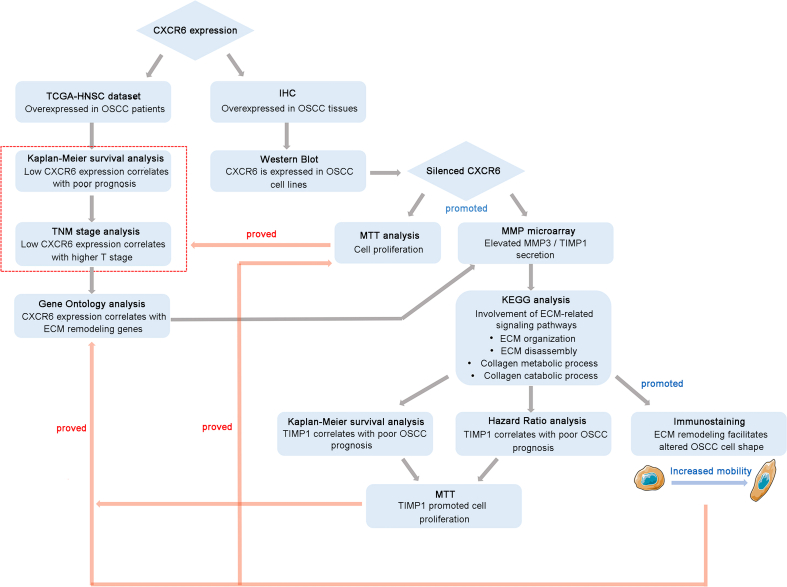# Low CXCR6 expression drives extracellular matrix remodeling and enhances cell proliferation in OSCC

**DOI:** 10.1016/j.gendis.2024.101213

**Published:** 2024-01-17

**Authors:** Yang Xun, Hua Yang, Yilong Ai, Honglin Li, Hua You, Fang Liu

**Affiliations:** Department of Basic Medicine and Biomedical Engineering, School of Medicine, Foshan University, Foshan, Guangdong 528000, China; Department of Basic Medicine and Biomedical Engineering, School of Medicine, Foshan University, Foshan, Guangdong 528000, China; Foshan Stomatological Hospital, Foshan, Guangdong 528000, China; Department of Basic Medicine and Biomedical Engineering, School of Medicine, Foshan University, Foshan, Guangdong 528000, China; Laboratory for Excellence in Systems Biomedicine of Pediatric Oncology, Department of Pediatric Hematology and Oncology, Children's Hospital of Chongqing Medical University, Chongqing 401122, China; Chongqing Key Laboratory of Pediatric Metabolism and Inflammatory Diseases, Ministry of Education Key Laboratory of Child Development and Disorders, National Clinical Research Center for Child Health and Disorders, Children's Hospital of Chongqing Medical University, Chongqing 401122, China; Centre for Translational Stem Cell Biology, Hong Kong 999077, China

Oral cancer, primarily oral squamous cell carcinoma (OSCC), is one of the most common cancer types worldwide. The incidence of OSCC continues to rise, with approximately 377,713 new cases and 177,757 deaths reported worldwide by 2020.^1^ Despite advancements in diagnosis and treatment, the 5-year survival rate for OSCC patients has remained at 60%–67% over the last two decades (SEER database: https://seer.cancer.gov/). This consistent rise in morbidity and lack of survival improvement have made OSCC a public concern.

Chemokines within the tumor microenvironment play an important role in inflammation-associated tumor development, invasion, and metastasis.^2^ Chemokine C-X-C receptor (CXCR) 6, and its ligand, CXCL16, are co-expressed in oral tissues, indicating a potential association with oral inflammation. However, the precise role of CXCR6 in OSCC development remains elusive. In this study, we discovered that CXCR6 is overexpressed in OSCC and is correlated with disease progression and unfavorable prognosis. Notably, CXCR6 drives OSCC cell proliferation by inducing the secretion of matrix metalloproteinase (MMP) 3 and tissue inhibitor of metalloproteinase (TIMP) 1, actively remodeling the extracellular matrix (ECM).

To investigate the role of CXCR6 in OSCC patients, we examined The Cancer Genome Atlas-Head and Neck Squamous Cell Carcinoma (TCGA-HNSC) dataset (*n* = 562) from the UCSC database (https://genome.ucsc.edu). The gene expression data for CXCR6 (ENSG00000172215) was extracted and filtered from “Primary Tumor” sources. Data analysis revealed substantial CXCR6 overexpression (*P* < 0.001) in OSCC tumor tissue compared with adjacent normal tissues (Fig. 1A). Notably, elevated CXCR6 expression among OSCC patients correlated with prolonged overall survival, with a median overall survival of 4.9 years, compared with 3 years for those with relatively low CXCR6 expression (hazard ratio = 0.672, *P* = 0.00387) (Fig. 1B). Further analysis was conducted to explore the relationship between CXCR6 and tumor TNM stages, revealing that OSCC patients with lower CXCR6 expression exhibited higher T stages, reflective of larger tumor volumes and, consequently, a less favorable prognosis (Fig. 1C). No significant correlation between CXCR6 expression and N or M stages was observed in OSCC patients (Fig. S1A, B).

**Figure 1:**
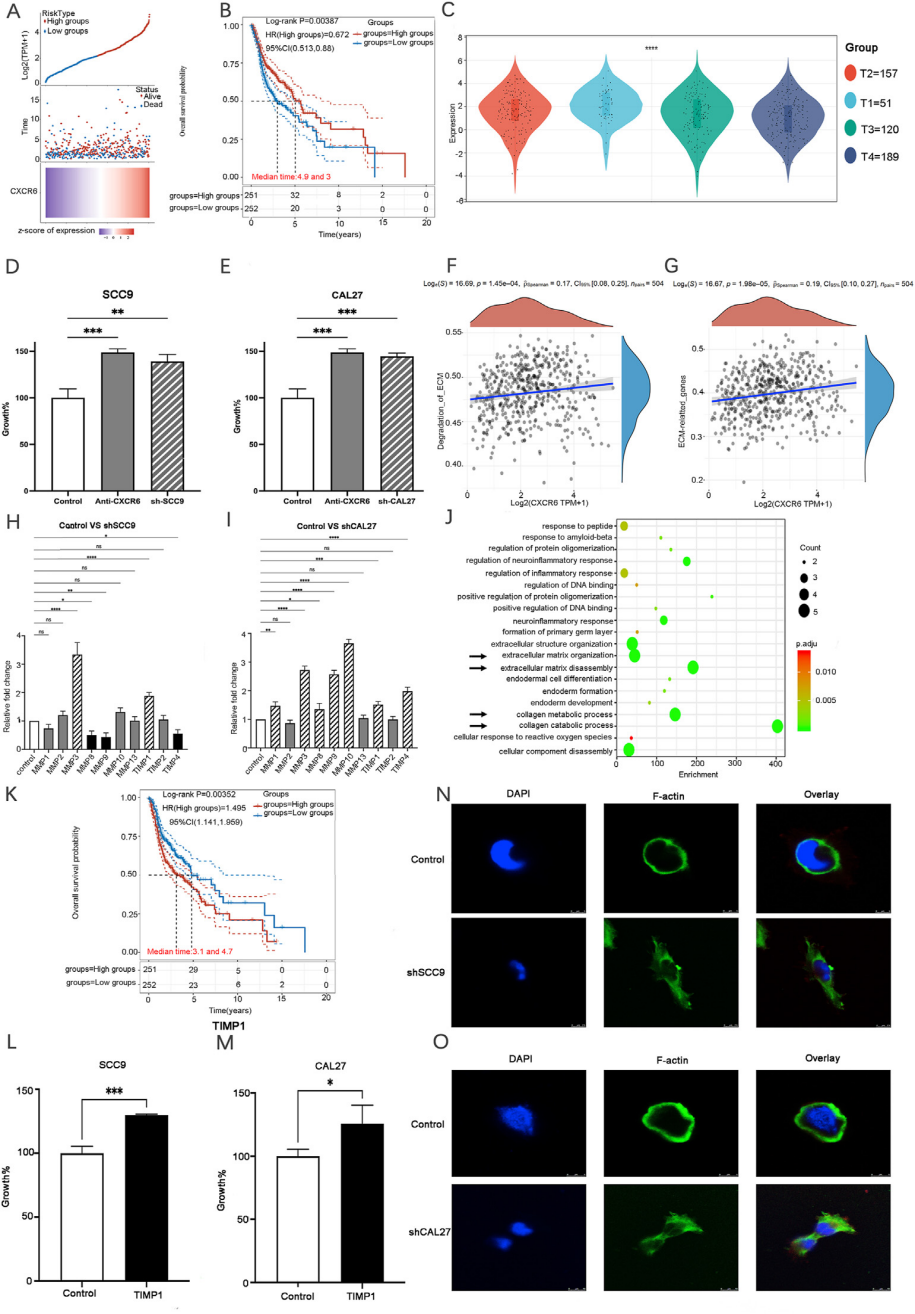
Low expression of CXCR6 promotes OSCC cell proliferation through TIMP up-regulation. **(A)** TCGA-HNSC dataset analysis of CXCR6 expression in OSCC tissues. The CXCR6 gene expression data extracted from 562 OSCC samples revealed significant overexpression of CXCR6 in tumor tissues compared with adjacent normal tissues (*P* < 0.001). **(B)** The impact of CXCR6 expression on the OS of OSCC patients. Kaplan–Meier survival analysis indicated that high CXCR6 expression correlated with prolonged OS compared with those with relatively low CXCR6 expression (4.9 years *vs.* 3 years, hazard ratio = 0.672, *P* = 0.00387). **(C)** Correlation between CXCR6 expression level and tumor T stages in OSCC patients. Lower CXCR6 expression level was associated with higher T stages, indicating poorer prognosis. **(D, E)** The impact of CXCR6 blockade on OSCC cell proliferation. Addition of anti-CXCR6 or CXCR6 knockdown significantly increased cell proliferation of SCC9 (D) and CAL27 (E) cell lines as assessed by MTT assay. **(F, G)** Gene Ontology (GO) and Kyoto Encyclopedia of Genes and Genomes (KEGG) analyses of the association between CXCR6 expression and cell responses. CXCR6 expression was closely associated with “degradation of ECM” (F) and “ECM-related genes” (G) (*P* < 0.05). **(H, I)** MMP and TIMP secretion in CXCR6 shRNA groups of OSCC cells. MMP microarray analysis showed significant elevation of MMP3 and TIMP1 secretion in the shRNA group of SCC9 (H) and CAL27 (I) cell lines (*P* < 0.01). **(J)** KEGG enrichment analysis of genes associated with the MMP microarray. Genes closely associated with the impact of CXCR6 blockade include those involved in extracellular matrix disassembly, extracellular matrix organization, and collagen metabolic/catabolic processes. **(K)** Kaplan–Meier survival analysis of the impact of TIMP1 expression on OSCC patients. TIMP1 had a significant effect on the survival of OSCC patients (*P* = 0.0035). **(L, M)** Effect of exogenous TIMP1 administration on OSCC cell proliferation. MTT assay demonstrated that the exogenous addition of TIMP1 significantly promoted the proliferation of SCC9 (L) and CAL27 (M) cells. **(N, O)** F-actin immunostaining of OSCC cells upon CXCR6 blockade. SCC9 (N) and CAL27 (O) cells underwent shape polarization and exhibited filopodia-rich appearance when CXCR6 was suppressed using shRNAs. CXCR6, chemokine C-X-C receptor 6; OSCC, oral squamous cell carcinoma; TIMP, tissue inhibitor of metalloproteinase; OS, overall survival; ECM, extracellular matrix; MMP, matrix metalloproteinase; shRNA, short hairpin RNA.

The expression of CXCR6 in OSCC tissue and cells was subsequently validated. Immunohistochemistry revealed significant CXCR6 expression in tongue squamous cell carcinoma compared with adjacent normal tissue (Fig. S1C, D). *In vitro*, immunocytochemistry demonstrated predominant CXCR6 localization on the cell membrane of OSCC cell lines SCC9 and CAL27 (Fig. S1E, F). Western blot results further confirmed stable CXCR6 overexpression in SCC9 and CAL27 cell lines, which was effectively suppressed by short hairpin RNAs (shRNAs) (Fig. S1G). Moreover, in both SCC9 and CAL27 cell lines, the addition of an anti-CXCR6 antibody or CXCR6 knockdown significantly increased cell proliferation rates after a 24-h incubation, as evidenced by MTT assay results (Fig. 1D, E). These findings aligned with the TCGA-HNSC dataset and Kaplan–Meier survival analysis, underscoring its clinical relevance and potential impact of CXCR6 on OSCC progression.

We further investigated the relationship between CXCR6 expression in OSCC cells and tumor progression. Gene Ontology (GO) analysis in OSCC revealed a strong association of CXCR6 expression with “degradation of ECM” (*P* < 0.001) and “ECM-related genes” (*P* < 0.001) (Fig. 1F, G). As MMPs are responsible for ECM degradation by breaking down collagen, fibronectin, and other ECM proteins, while TIMPs serve as inhibitors of MMPs to maintain ECM integrity, we examined the secretions of MMPs and TIMPs from OSCC cells using MMP microarray. The outcomes revealed a significant increase in MMP3 and TIMP1 in the shRNA group of both SCC9 (Fig. 1H) and CAL27 cell lines (Fig. 1I) (*P* < 0.001). Other MMPs exhibited subtle alterations or inconsistent trends between the two cell lines. This result reflects a dynamic ECM environment where MMP3-mediated ECM degradation may trigger a cascade of events leading to TIMP1 expression, aimed at preserving ECM integrity. This imbalance in the ECM structure ultimately leads to elevated tumor cell mobility and progression. A similar phenomenon has been reported in colorectal cancer, where the simultaneous oversecretion of MMP3 and TIMP1 positively correlated with tumor staging.^3^

Kyoto Encyclopedia of Genes and Genomes (KEGG) enrichment analysis of genes associated with the MMP microarray was then performed. The results suggested significant differences in ECM-related signaling pathways, including ECM organization, disassembly, and collagen metabolic/catabolic processes in the shCXCR6 group compared with the control group (Fig. 1J), implying ECM remodeling. The impact of MMP secretion on the overall survival and hazard ratio of OSCC patients was further analyzed. MMP3 showed no substantial effect on survival (*P* = 0.56) (Fig. S1H, I). In contrast, TIMP1 expression status exerted a significant influence and correlated with poorer prognosis in OSCC patients (*P* = 0.0035, hazard ratio = 1.50, 95% confidential interval:1.14–1.96), as demonstrated by Kaplan–Meier survival analysis (Fig. 1K).

Subsequently, the impact of exogenously administrated TIMP1 on OSCC cell proliferation was assessed, revealing its substantial promotional ability, as indicated in the MTT results (Fig. 1L, M). This further confirmed the involvement of TIMP1 in mediating ECM remodeling and OSCC progression. TIMP1 may possess additional functions beyond ECM remolding, including inducing growth factor accumulation in the ECM, activating tumor cell growth pathways, and modulating immune responses via cell surface receptors, thereby promoting cell proliferation and tumor progression. However, the precise singling pathways and molecular mechanisms driving this phenomenon in OSCC require in-depth investigation.

The study then evaluated the effect of ECM remodeling on OSCC cell division and polarization using immunostaining (Fig. 1N, O). In the control group, both SCC9 and CAL27 cell lines displayed polygonal and flat shapes, indicating a densely packed structure with limited cell proliferation ability. Conversely, the CXCR6 low-expression group in both cell lines exhibited a filopodia-rich and lamellipodial shape with higher polarity. These morphological changes are closely associated with the asymmetric distribution of cellular components and an increased capacity for cell mobility, facilitating growth and movement within the ECM. Remodeled ECMs can significantly impact cell spreading by altering stiffness, cell adhesion site availability, and constraints for cell movement. Consequently, these alterations influence mechanosensitivity and the ability of cells to proliferate.^4^ Collectively, we hypothesize that decreased CXCR6 expression induces elevated MMP3 and TIMP1 secretion, resulting in a more dynamic ECM. This, in turn, triggers mechanosensitive pathways leading to changes in cell shape, mobility, adhesion, and ultimately, cell proliferation, and tumor progression.

In this study, we report CXCR6 overexpression in OSCC tissues and cells. Decreased CXCR6 expression significantly impacts the prognosis of OSCC patients by promoting cell proliferation, similar to the observation in hepatocellular carcinoma.^5^ CXCR6 is associated with ECM remodeling via up-regulation of MMP3 and TIMP1. This drives OSCC cells into a more motile filopodia-rich and lamellipodial shape with higher polarity, enhancing their proliferation capability (Fig. S2).

CXCR6 exhibits a diverse expression pattern within tumor tissues, influenced by complex factors and feedback mechanisms within the tumor microenvironment. These factors may include stimulation by pro- or anti-tumor factors and metabolites, and infiltration of CXCR6-expressing immune cells with potential pro- or anti-tumor effects. As a membrane receptor, CXCR6 initiates downstream signaling pathways upon interacting with CXCL16. Disruptions in this interaction or alterations in signaling pathways can impact tumor cell growth and contribute to tumor progression. Epigenetic modifications may also lead to abnormal CXCR6 gene expression and cell growth. Investigating the underlying mechanisms and the physical alterations of the ECM structure resulting from the impact of CXCR6 requires further *in vitro* and *in vivo* investigations. While the precise mechanisms are intricate, our findings lay the groundwork for understanding the multifaceted role of CXCR6 in OSCC progression, offering new avenues for future research aimed at enhancing OSCC treatment strategies.

## Ethics declaration

Ethical approval was obtained from the Ethics Committee of Foshan University (approval number Medical-2022003), and written informed consents were obtained from all participants. All authors agreed to publish this manuscript.

## Author contributions

Y.X and H. Yang were the principal investigators for the study. Y.X., H. Yang, and F.L. conceived the study and carried out a major part of the project. H.L. performed figure editing according to the journal's request. Y.X. and H. Yang collected the clinical and experimental data and made significant improvements to the preliminary data. Y.X., H. Yang, and H. You wrote the manuscript. A.Y. and F.L. contributed to a critical review of the manuscript. The authors read and approved the final manuscript.

## Conflict of interests

All authors declared no conflict of interests.

## Funding

This study was supported by the 10.13039/501100001809National Natural Science Foundation of China (No. 82203213, 82203844, 81911530169), Innovation Technology Commission Funding (Health@InnoHK) in Hong Kong, China, Basic and Applied Basic Research Project of Guangdong Province, China (No. 2020A1515111201, 2019A1515110495), and University Special Innovative Research Program of Department of Education of Guangdong Province, China (No. 2020KQNCX073).

## Data availability

The data sets used and analyzed during the current study are available from the corresponding author upon reasonable request.

## Figures and Tables

**figs1:**
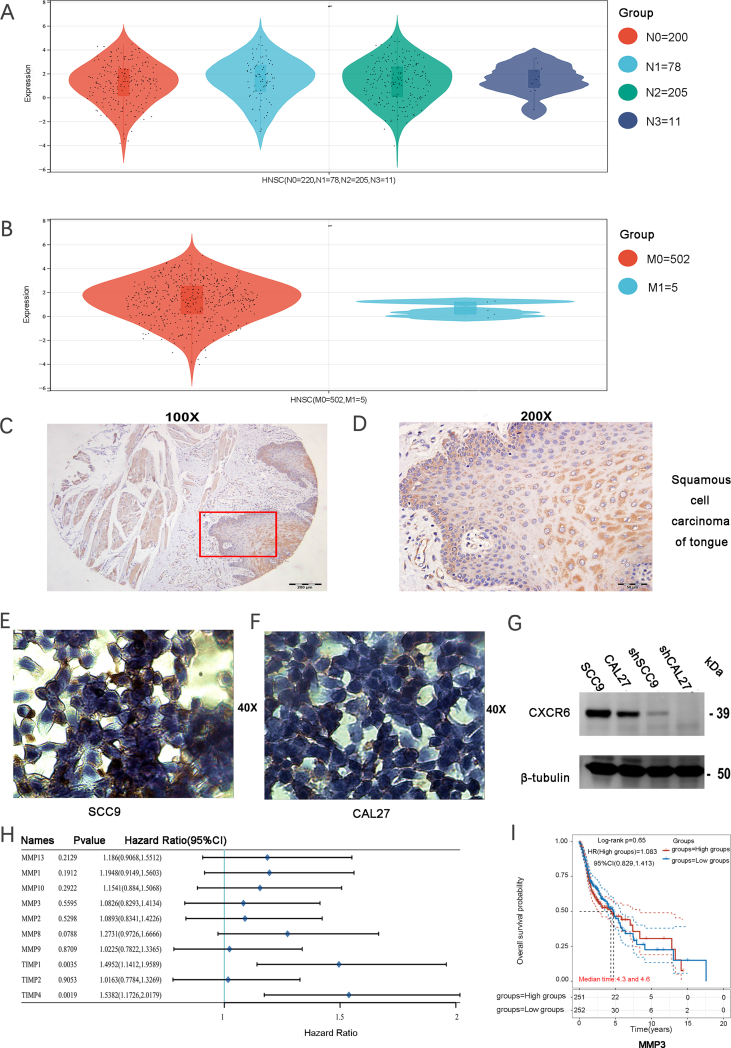


**figs2:**