# Supplementary material for: Low CXCR6 expression drives extracellular matrix remodeling and enhances cell proliferation in OSCC
Source: Genes Dis. 2024 Jan 17;12(1):101213. doi: 10.1016/j.gendis.2024.101213 (PMC11416661; doi:10.1016/j.gendis.2024.101213)
Supplement: Multimedia component 1 [file mmc1.docx]

**Supplementary data**

**Materials & Methods**

**Clinical Data Analysis**

# RNA-sequencing expression profiles (level 3) and corresponding clinical information pertaining to CXCR6 were acquired from the TCGA dataset (https://portal.gdc.com). A total of 503 OSCC patients were included and subsequently categorized into low- (n = 251) and high- (n = 252) expression groups based on the median CXCR6 expression. The expression distribution of CXCR6 gene in both tumor and normal tissues were examined using R software (Foundation for Statistical Computing, 2020) version 4.0.3. In instances where not explicitly specified, the comparison of two-group data was performed using the Wilcox test. To compare the differences in survival between these groups, log-rank test was employed, accompanied by the generation of Kaplan-Meier survival curves. *P* values and hazard ratio (HR) with 95% confidence interval (CI) were generated via log-rank tests and univariate Cox proportional hazards regression analysis. All the analysis methods, along with relevant R packages, were implemented within R software version 4.0.3, with statistical significance set at *P* < 0.05.

# To explore correlations between genes and pathways, R software package GSVA with the selected parameter method='ssgsea'. The assessment of correlations between gene expression and pathway scores was conducted using Spearman correlation analysis.

**Cell Lines and Culture**

# OSCC cell lines SCC9 and CAL27 were purchased from the American Type Culture Collection (ATCC, Manassas, VA, USA). The cells were cultured in a 5% CO_2_ incubator at 37°C in DMEM/F-12 medium (Gibco, New York, USA) with 10% fetal bovine serum (FBS) (Gibco, New York, USA) and 1% 100 μg/ml streptomycin and 100 units/ml penicillin (Beyotime, Shanghai, China). Cells were passaged when they reached 80–90% density, and logarithmically growing cells were used for subsequent studies.

**Immunohistochemical Staining and Immunocytochemistry Staining**

# Oral squamous carcinoma tissue microarrays were purchased from Shanghai OUTDO Biotech Co. Ltd (Shanghai, China). Immunohistochemical staining was used to detect CXCR6 protein expression in two pairs of OSCC cancer tissues and adjacent normal tissues. Tissue sections were deparaffinized, hydrated, and treated with hydrogen peroxide to remove endogenous peroxidase for antigen retrieval. Anti-CXCR6 antibodies (Invitrogen, New York, USA) were applied and incubated for 1 hour at 37°C, followed by 30 minutes cooling at room temperature. After rinsing with PBS, secondary antibodies (Boster, China) were applied and incubated for additional 20 minutes. Color development was achieved using the DAB kit (Boster, China) and examined under a microscope. Hematoxylin was used for counterstaining acidophilic structures turning blue. The section was then dried and mounted with neutral resin.

# For immunocytochemistry staining, OSCC cells were fixed with 4% paraformaldehyde for 2 hours, rinsed three times with prewarmed PBS, and counterstained with hematoxylin to visualize acidophilic structures turning blue. Once translucent, the sections were mounted with neutral glue.

**CXCR6 Gene Knockdown**

# To silence the expression of the CXCR6 gene, a short hairpin RNA (shRNA) sequence was designed and purchased from Genepharma (Shanghai, China). The lentivirus was harvested 48 hours after co-transfecting the designated plasmids, along with psPAX2 and pMD2.G, or their corresponding empty vectors, into HEK-293T cells. Lipofectamine 3000 (Invitrogen, California, USA) was employed for transfection. The target cells were infected with the filtered lentivirus, supplemented with 6 µg/ml Polybrene (Sigma, Missouri, USA) for a duration of 48 hours.

**Western Blot Analysis**

# Total protein was extracted using RIPA lysis buffer and quantified using a BCA protein assay kit. Protein samples (40 μg) were resolved via SDS-PAGE and subsequently transferred onto PVDF membranes (Millipore, Massachusetts, USA). After 2 hours of blocking with 5% nonfat milk, the membranes were incubated with anti-CXCR6 antibody (Invitrogen, New York, USA) for 18 hours at 4 °C, followed by 1 hour at room temperature with a rabbit secondary antibody (Invitrogen, New York, USA). Membrane imaging was performed using the Odyssey Sa Imaging System (LI-COR Biosciences, USA). β-tubulin was employed as a loading control.

**Cell Proliferation Assays**

# OSCC cells were cultured in 96-well plates at a density of 2 × 10^3^ cells per well and incubated for at 37°C in a 5% CO_2_ incubator for 24 hours. The cells were subjected to experimental treatment, including anti-CXCR6 (Invitrogen, New York, USA), TIMP1 (Abcam, Cambridge, UK), or relevant controls, and incubated for further 24 hours. Subsequently, MTT reagent (Sigma-Aldrich, Missouri, USA) was added to each well, followed by a 2–4-hour incubation at 37 °C. Dimethyl sulfoxide (DMSO) was then applied and the optical density (OD) was measured at 450 nm using a microplate reader (ThermoFisher Scientific, Massachusetts, USA). The relative cell viability was calculated, all data represent the mean of at least three independent experiments.

**MMP Microarray Analysis**

# After 24 hours of culture, supernatants of OSCC cells were collected. The expression levels of matrix metalloproteinases (MMP1, MMP2, MMP3, MMP8, MMP9, MMP10, MMP13) and tissue inhibitor of metallopeptidases (TIMP1, TIMP2, and TIMP4) were assessed using a specialized MMP microarray kit (RayBiotech, Inc., China). The microarray kit was prepared according to the manufacturer’s instructions.

**Immunofluorescent Staining and Confocal Imaging**

# The cytoskeleton and nucleus were stained using phalloidin (Invitrogen, New York, USA) and 4′,6-diamidino-2-phenylindole (DAPI), respectively. The samples were initially fixed with immunohistochemically fixed fluid (Beyotime, China) for 30 min, followed by three rinses with prewarmed PBS. Subsequently, the samples were blocked with 0.1% bovine serum albumin solution for 1 hour at 37°C, followed by incubation with DAPI (1:400 dilution) and Alexa Fluor Phalloidin 488 conjugate (1:200 dilution) for 2 hours at 37°C. Imaging was conducted using a laser scanning confocal microscope (Leica SP8, USA). In line with the imaging measurement schematic, a semiconductor laser of 488 nm was selected as the excitation source.

# Statistical Analysis

# Statistical analyses were conducted using online resources available at http://SangerBox.com/Tool and https://www.cbioportal.org/. Unpaired t-tests were employed for comparison between two groups, while multiple-group comparisons were executed using one-way ANOVA. Significance levels denoted as followed: * for *P <* 0.05, ** for *P* < 0.01, *** for *P* < 0.001, and **** for *P* < 0.0001, indicating statistical significance.


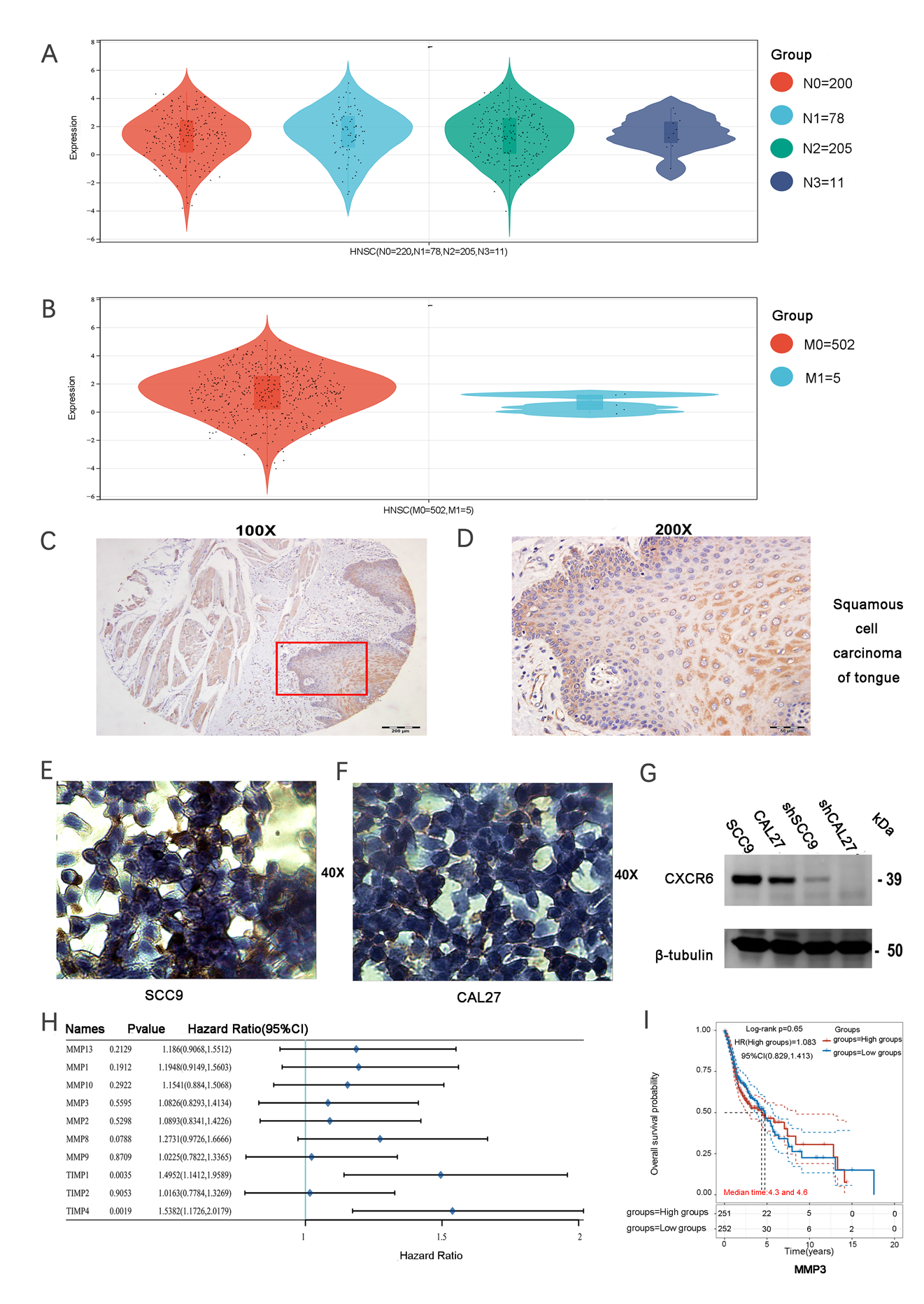
**Figure S1**

**A-B. Relationship between CXCR6 expression and tumor N and M stages**. In OSCC patients, no significant correlation between CXCR6 expression and either N (A) or M (B) stages.

**C-D. CXCR6 expression in squamous cell carcinoma of tongue.** CXCR6 exhibited strongly expression in tumor tissues, as depicted by the brown color in immunohistochemistry staining. Image C represents 100× magnification, while image D represents 200× magnification.

**E-F. CXCR6 expression on OSCC cells.** CXCR6 was predominantly expressed on the cell membrane of OSCC cell lines SCC9 (E) and CAL27 (F) as revealed by immunochemistry staining.

**G. Expression of CXCR6 and CXCR6 ablated with shRNAs in OSCC cells.** Western blot analysis demonstrated stable CXCR6 overexpression in SCC9 and CAL27 cells, as well as its ablation using short hairpin RNAs (shRNAs).

**H. Hazard Ratio analysis of MMPs/TIMPs expression in OSCC patients.** TIMP1 exhibited a significant association with the survival of OSCC patients (*P* = 0.0035).

**I. Kaplan-Meier survival analysis of the impact of MMP3 expression on OSCC patients.** MMP3 did not significantly affect the survival of OSCC patients (*P* = 0.56)

**
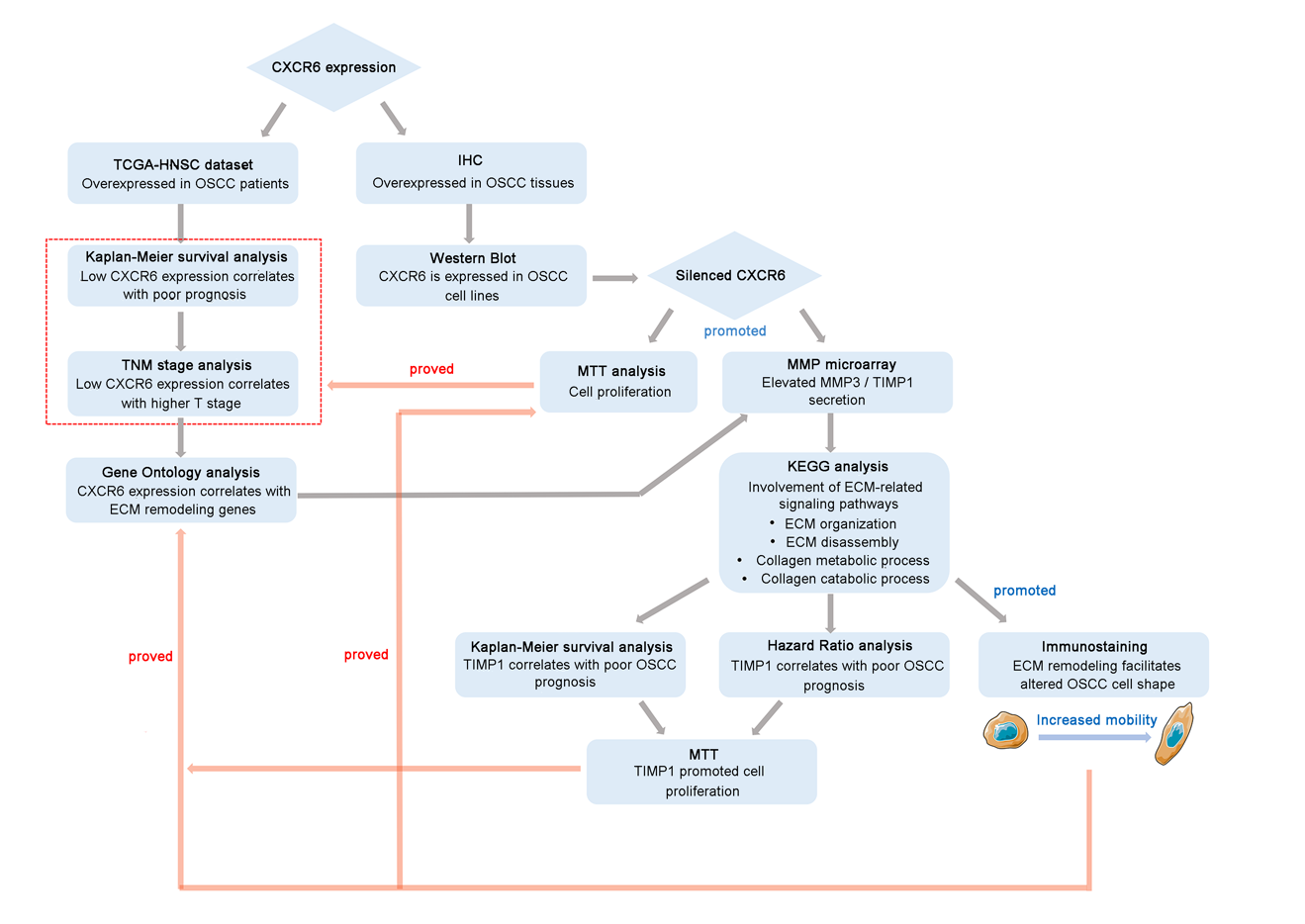
**

**Figure S2**. The experiment flowchart.
